# Case report: Different outcomes of two cases of relapsed/refractory T cell acute lymphoblastic leukemia treated with anti-CD7 chimeric antigen receptor T cells bridging to allogeneic hematopoietic stem cell transplantation: from curative promise to fatal risk

**DOI:** 10.3389/fonc.2026.1766948

**Published:** 2026-01-29

**Authors:** Zhijuan Pan, Yanru Guo, Ying Zhang, Shuting Chang, Jiajia Sun, Zhiping Guo, Yiqun Zhang

**Affiliations:** Department of Hematology, Peking University First Hospital Taiyuan Hospital (Taiyuan Central Hospital, The Ninth Clinical School of Shanxi Medical University), Taiyuan, Shanxi, China

**Keywords:** acute lymphoblastic leukemia, anti-CD7 chimeric antigen receptor T cells, hematopoietic stem cell transplantation, inflammatory factors, refractory, relapsed

## Abstract

Consolidative allogeneic hematopoietic stem cell transplantation (allo-HSCT) after chimeric antigen receptor (CAR) T-cell therapy is an emerging modality in hematologic malignancies. Knowledge regarding the optimal interval and pretreatment regimen between CAR T-cell therapy and allo-HSCT remains limited. Here, we report two cases of confirmed relapsed/refractory (R/R) T-cell acute lymphoblastic leukemia (T-ALL) treated with autologous anti-CD7 CAR T cells infusion bridging to allo-HSCT. Case 1 had a poor prognosis due to grade 3 cytokine release syndrome (CRS), infection, drug-related organ toxicity, hyperacute graft-versus-host disease (GVHD), and transplant-associated thrombotic microangiopathy (TA-TMA). In contrast, case 2 demonstrated a favorable course, marked by effective inflammation control, complete recovery following CAR T-cell therapy, and timely transplantation. These cases indicate that anti-CD7 CAR T-cell therapy represents a promising therapeutic strategy for R/R T-ALL. However, its integration with allo-HSCT constitutes a high-risk clinical approach that requires careful and individualized management.

## Introduction

Adult T cell acute lymphoblastic leukemia (ALL) is an aggressive disease that frequently presents with a high tumor burden, leukocytosis, and central nervous system involvement, and comprises approximately 25% of all adult ALL cases (1, 2). Although the complete remission (CR) rate after induction chemotherapy is greater than 90%, approximately 40% of patients experience disease relapse (3, 4). The overall outcome of relapsed/refractory (R/R) T-ALL in adults is extremely poor, with <10% of patients surviving 5 years after diagnosis (5, 6).

CD7 is highly expressed in T-ALL, and CD7 chimeric antigen receptor (CAR) T-cell therapies have emerged as a safe and effective salvage strategy for R/R T-ALL (7–9). In addition, CD7 CAR T-cell infusion bridging to allogeneic hematopoietic stem cell transplantation (allo-HSCT), which is a potentially curative treatment, can provide deep and durable responses in R/R T-ALL (10, 11).

At present, there is no consensus on the timing or components of the conditioning regimen for allo-HSCT following CAR T-cell treatment. Moreover, only a limited number of studies with small case numbers have focused on the association between CD7 CAR T-cell therapy and transplantation-associated complications, including graft-versus-host disease (GVHD) and thrombotic microangiopathy (TA-TMA). Therefore, treatment of R/R T-ALL with CD7 CAR T cells bridging to allo-HSCT still requires further investigation. Here, we report two cases with similar disease features who underwent allo-HSCT following CD7 CAR T-cell infusion but demonstrated markedly different outcomes, in order to evaluate the efficacy and safety of this therapeutic strategy. These two cases may provide insight into the sequential use of CD7 CAR T-cell therapy and allo-HSCT in R/R T-ALL.

## Case presentation

### Case 1

**Figure 1:**
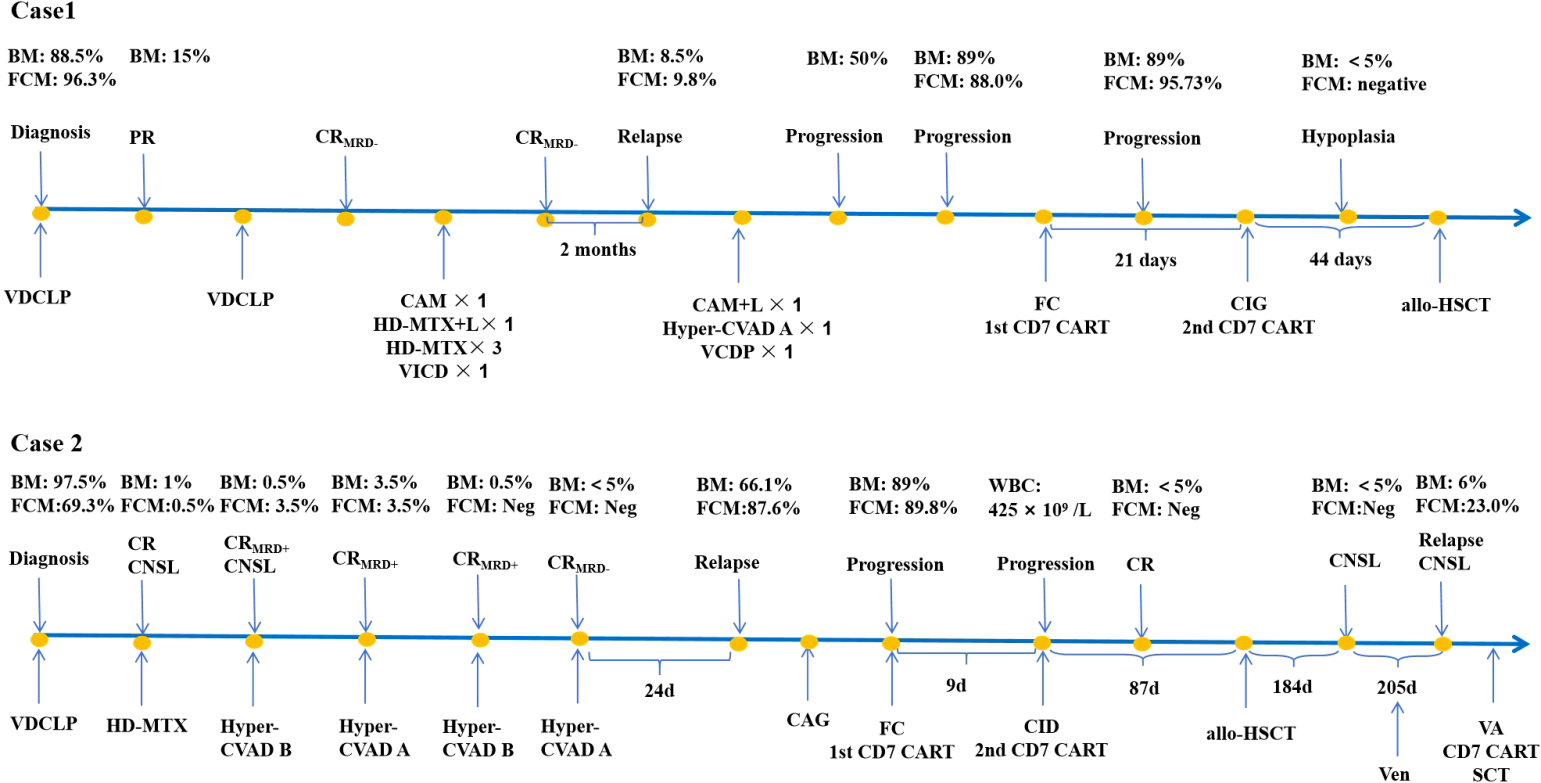
Clinical course of the patients. PR, partial remission; CR, complete remission; FCM, flow cytometry; BM, bone marrow; CNSL, central nervous system leukemia; MRD, minimal residual disease. VDCLP, vinodixin 4mg d1/8/15/22, daunorubicin 40mg/m^2^/d d1/8/15/22, cyclophosphamide 750mg/m^2^/d d1/15, pegaspargase 3750IU d8/22, dexamethasone 10mg d1–14 and 5mg d15-28. CAM ± L, cyclophosphamide 750mg/m^2^/d d1/8, cytarabine 100mg/m^2^/d d1–3 and d8-10, 6-mercaptopurine, 60mg/m^2^/d d1-7, pegaspargase 3750IU d2. HD-MTX ± L, high-dose methotrexate 3g/m^2^ d1, pegaspargase 3750IU d3. Hyper-CVAD A, cyclophosphamide 300mg/m^2^ q12h d1-3, vinodixin 4mg d4/11, doxorubicin 50mg/m^2^ d4, dexamethasone 40mg/d d1-4/11-14. Hyper-CVAD B, high-dose methotrexate 1g/m^2^ d1, cytarabine 3g/m^2^ q12h d2-3. FC, fludarabine 30mg/m^2^ d-4 to -2, cyclophosphamide 500mg/m^2^ d-3 to -2. CIG, cytarabine 20mg/m^2^ q12h d1-7, idarubicin 5mg/m^2^ d1-3, granulocyte colony‐stimulating factor (G‐CSF) 200μg/m^2^/d d0-7. CAG, cytarabine 20mg/m^2^ q12h d1-7, aclarubicin 20 mg d1-4, G‐CSF 200μg/m^2^ d0-7. CID, cyclophosphamide 200mg/m^2^/d d1-3, idarubicin 10mg/m^2^ d1-3, dexamethasone 7.5mg d1-5. AV, azacitidine 100mg d1-7, venetoclax 400mg d1-14.

A 39-year-old male patient presented to a local hospital in December 2019 with flu-like symptoms lasting 2 weeks. Blood routine examination revealed the following results: white blood cell count (WBC), 84.82 × 10^9^/L; hemoglobin (Hb), 74 g/L; and platelet count (Plt), 14 × 10^9^/L. Bone marrow (BM) examination showed blast cells accounting for 88.5% of the total cell population. Further immunophenotypic analysis by flow cytometry (FCM) revealed that blast cells comprising 96.3% of the population, expressing CD7, CD34, CD38, CD99, cyCD3, cCD3, and sCD3. Karyotype analysis revealed a normal karyotype (46, XY) [20]. The patient was diagnosed with mature T-ALL and classified as the high-risk group based on his age >35 years. Initially, he received two cycles of vinodixin, daunorubicin, cyclophosphamide, pegaspargase, and dexamethasone (VDCLP) and achieved complete remission with minimal residual disease negativity (CR_MRD_−), as verified by FCM. Subsequently, the patient received six cycles of consolidation chemotherapy (**Figure 1**) and four lumbar punctures with intrathecal chemotherapy, with no abnormal white blood cells detected in the cerebrospinal fluid (CSF). Unfortunately, shortly after the final consolidation cycle, the disease relapsed, with BM morphology revealing 8.5% blasts on 30 January 2021.

The patient then received three courses of salvage chemotherapy. However, the disease continued to progress, with 88% lymphoblasts detected in the BM. The leukemic blasts were immunophenotypically characterized as cCD3, CD7, CD99, CD5^dim, and cTdT positive; CD34 partially positive; and CD3, CD4, CD8, and CD2 negative. Thus, the leukemia was refractory to salvage chemotherapy, and the relapsed blasts exhibited immunophenotypes similar to those of the primary blasts. Therefore, CD7 CAR T-cell therapy was introduced.

**Figure 2:**
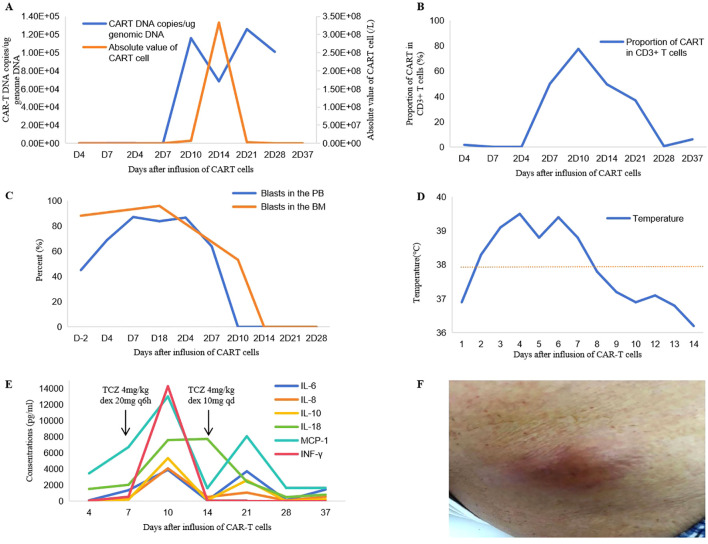
Changes in case 1 indicators in case 1 after CD7 CAR T-cell therapy. Trends in CAR T DNA copies and absolute CAR T-cell counts in peripheral blood (PB) **(A)**. Proportion of CAR T cells among CD3^+^ T cells **(B)**. Blasts in BM and PB **(C)**. Temperature changes **(D)**. Levels of inflammatory cytokines **(E)**. Skin and soft tissue infection in the anterior iliac region **(F)**. 2D, days after the second CAR T-cell infusion. TCZ, tocilizumab; dex, dexamethasone.

Peripheral blood (PB) of the patient was collected to prepare the autologous humanized naturally selected anti-CD7 CAR (NS7CAR) T cells. The CAR T cells were engineered by Hebei Senlangio Technology Co., Ltd. (Shijiazhuang, China). Lymphodepleting chemotherapy with the FC regimen (fludarabine 30 mg/m², days −4 to −2; cyclophosphamide 500 mg/kg, days −3 to −2) was initiated on May 27 2021 (day −4). On day 0, the patient received an infusion of CD7 CAR T cells at a dose of 1 × 10^6^ cells/kg. However, MRD in the BM increased to 95.73% on day 18 after CAR T-cell infusion. After one cycle of cytarabine, idarubicin, and granulocyte colony-stimulating factor (CIG) for tumor reduction, the patient received a second CD7 CAR T-cell infusion on June 21 2021 at a dose of 2 × 10^6^ cells/kg. As expected, with the expansion of the reinfused CAR T cells (**Figures 2A, B**), the leukemic blasts gradually decreased (**Figure 2C**).

Following the second CD7 CAR T-cell infusion, a series of clinical changes occurred. Scattered rashes appeared on day 2, and the lesions gradually expanded. Fever developed on day 2, peaked at 39.5 °C, and persisted for 1 week (**Figure 2D**). On day 8, hypotension occurred, requiring norepinephrine or dopamine for blood pressure support. Low percutaneous oxygen saturation was detected, and oxygen inhalation at 10 L/min via mask was initiated. Peak serum levels of interleukin-6 (IL-6; 297-fold above the baseline) and monocyte chemoattractant protein-1 (MCP-1) were detected on day 10 after the infusion (**Figure 2E**). In addition, the patient developed some signs of organ toxicity, including elevated transaminases, serum creatinine, lactate dehydrogenase, and prolonged activated partial thromboplastin time (**Supplementary Figure S1**). Immune effector cell-associated neurotoxicity syndrome (ICANS), with an ICE score of 8 (the patient was unable to count backward from 100 by 10 or write a standard sentence), was observed on day 13. According to the ASTCT grading criteria for CRS and CRES (12), grade 3 cytokine release syndrome (CRS) and grade 1 CAR T-cell–related encephalopathy syndrome (CRES) were diagnosed. Accordingly, tocilizumab (anti–IL-6 receptor monoclonal antibody; 4 mg/kg once daily on days +8 and +14 days), dexamethasone (10 mg once daily on day +7; 10 mg every 6 h on days +8 to +9; 10 mg every 8 h on day +10; 10 mg every 12 h on day +11; and 10 mg once daily on days +13 to +14), and supportive care were administered. By day 15, the patient’s vital signs were stable, and the symptoms of ICANS had completely resolved, and the symptoms and indicators of CRS were gradually improving. On day 25, the patient’s WBC count remained close to zero, and unfortunately, localized skin and soft tissue infection developed in the anterior iliac region (**Figure 2F**).

**Figure 3:**
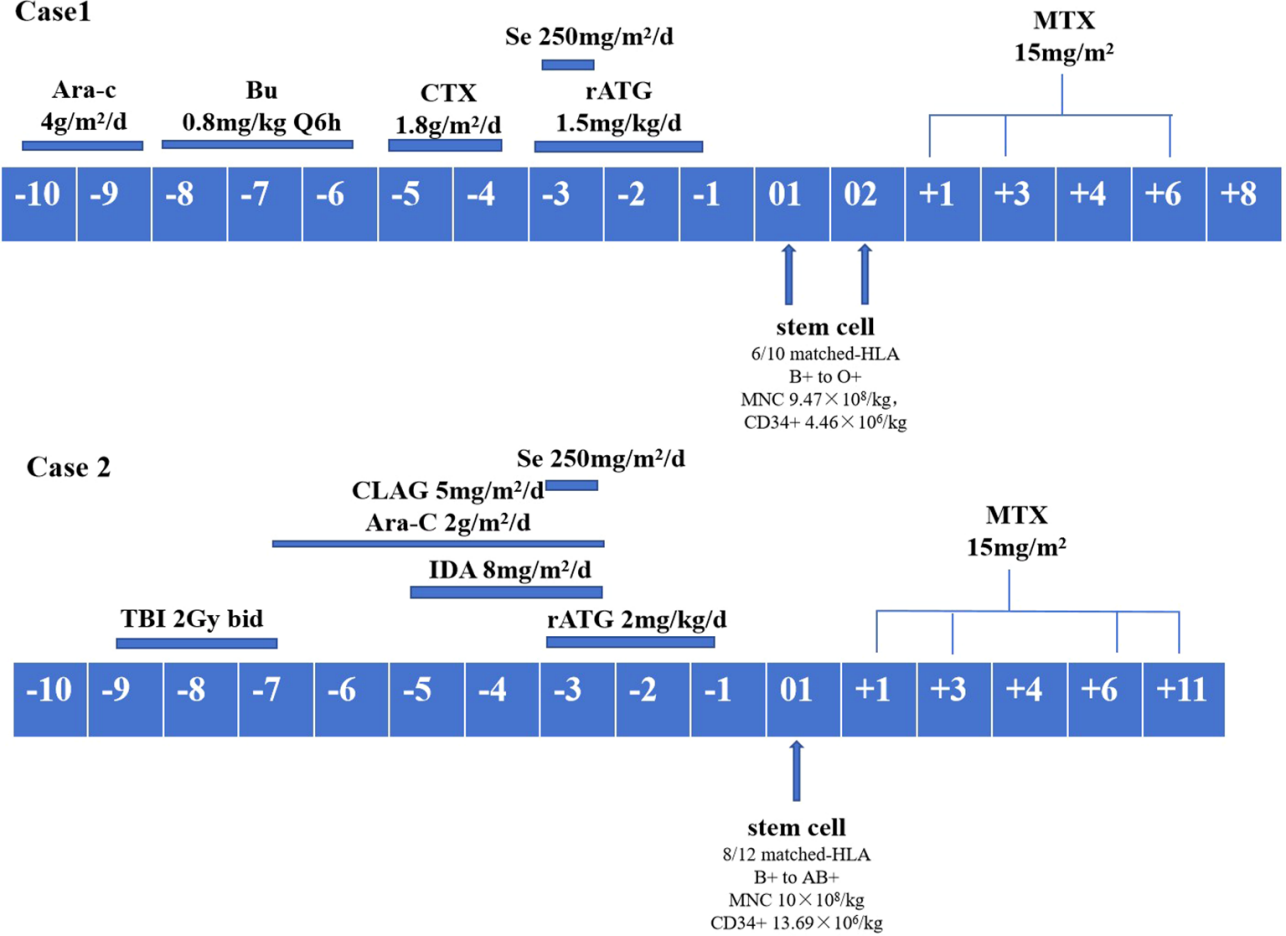
Conditioning regimens. Ara-C, cytarabine; Bu, busulfan; CTX, cyclophosphamide; Se, semustine; rATG, anti-thymocyte globulin, rabbit; MTX, methotrexate; TBI, total body irradiation; CLAG, cladribine; IDA, idarubicin.

Given the severe bone marrow suppression, refractoriness to platelet transfusion, dependence on blood transfusions, and skin and soft tissue infection with little chance of improvement without reestablishment of hematopoiesis, allo-HSCT (6/10 HLA-matched related donor, son-to-father transplantation) was recommended as an intervention to achieve disease control. Accordingly, on day 34 after the second CAR T-cell infusion, immediately following improvement of CRS and CRES and restoration of organ function, the patient received myeloablative busulfan plus cyclophosphamide (BU+CY) conditioning (**Figure 3**). Anti-thymocyte globulin (ATG, rabbit), cyclosporine A and methotrexate were administered for GVHD prophylaxis.

The patient still had a generalized rash when the conditioning regimen was initiated, and the absolute count of CD7 CAR T cells in the peripheral blood on day −7 was 1.61 × 10^4^/L. New neuropsychiatric symptoms, including hallucinations and incoherent speech, as well as cardiac insufficiency, developed from day −5, and symptomatic supportive treatment was provided. The patient’s vital signs remained stable prior to the transplantation. However, he developed fever on the first day of stem cell infusion and diarrhea (up to 2010 g/day) on the second day, which was considered as hyperacute gastrointestinal GVHD. The cyclosporine A dose was adjusted to achieve therapeutic concentrations, and mycophenolate mofetil (MMF), methylprednisolone, and basiliximab were administered. Diarrhea subsequently improved, but the patient developed extensive skin desquamation and peeling, along with elevated bilirubin levels.

Unfortunately, the patient gradually developed worsening neuropsychiatric symptoms, including a suspected seizure episode and coma, as well as persistent fever peaking at 42 °C that was unresponsive to antibiotics. On day +3 after allo-HSCT, the serum sC5b-9 level was 512 ng/mL, indicating a high risk of transplant-associated thrombotic microangiopathy (TA-TMA). Subsequently, red cell fragments were detected on peripheral blood smear. Additional abnormal findings included elevated levels of serum lactate dehydrogenase (LDH) and serum creatinine levels, increased requirements for red blood cell transfusion, continued ineffective platelet transfusion refractoriness, and proteinuria (2+). TA-TMA was diagnosed and treated with rituximab, eculizumab and supportive care. Despite these interventions, the patient’s clinical condition did not improve, and multiple organ functions continued to deteriorate. Hemodynamic instability developed on day +6 after transplantation. The patient was discharged on day +8 and died on the same day.

### Case 2

A 17-year-old male presented with a 10-day history of swollen lymph nodes and poor appetite in July 2020. Blood cell counts revealed WBC of 103.34 × 10^9^/L, Hb of 147 g/L, and Plt of 38 × 10^9^/L. Bone marrow smears showed lymphoblastic cells accounted for 97.5%. Immunophenotyping identified 69.3% of abnormal T cells expressing CD7, CD4, CD5, sCD3, CD99, CD2, CD38, and cCD3, with partial expression of CD1a. Karyotype analysis revealed 46, XY, der (1;7)(p22;p13) [20]. Genetic testing identified NOTCH1 mutation. The patient was diagnosed with T-ALL and classified as the high risk group based on WBC count >100 × 10^9^/L. The patient received induction therapy with vinodixin, daunorubicin, cyclophosphamide, pegaspargase, and dexamethasone (VDCLP) and achieved CR with MRD positivity, followed by one cycle of high-dose methotrexate (HD-MTX), two cycles of high-dose methotrexate plus cytarabine (hyper-CVAD B), and two cycles of cyclophosphamide, vinodixin, doxorubicin, and dexamethasone (hyper-CVAD A) as consolidation therapy (**Figure 1**). The patient maintained CR but never achieved MRD negativity in the BM by FCM during induction and consolidation therapy. Leukemic cells were detected in the CSF during the first course of consolidation chemotherapy, and the patient was diagnosed with central nervous system leukemia (CNSL). After one lumbar puncture with intrathecal chemotherapy, leukemic cells became undetectable in the CSF. Thereafter, the patient received additional lumbar punctures and intrathecal chemotherapy.

Unfortunately, one month after the final consolidation cycle, the patient experienced relapse, with WBC increasing to 231 × 10^9^/L. Leukemic blasts accounted for 87.6% of BM cells and were immunophenotypically characterized as CD99^bri, CD7, cTdT, CD2, CD5, CD34/CD1a, and CD3^dim positive, and CD56, CD4, and CD8 negative. However, the patient failed to achieve second complete remission after one cycle of salvage chemotherapy with cytarabine, aclarubicin, and granulocyte colony-stimulating factor (CAG). Because relapse occurred within one year, so the leukemia in the patient was considered relapsed and refractory. Given the high expression of CD7 on leukemic blasts, CD7 CAR T-cell therapy was recommended to achieve remission and bridge to allo-HSCT.

**Figure 4:**
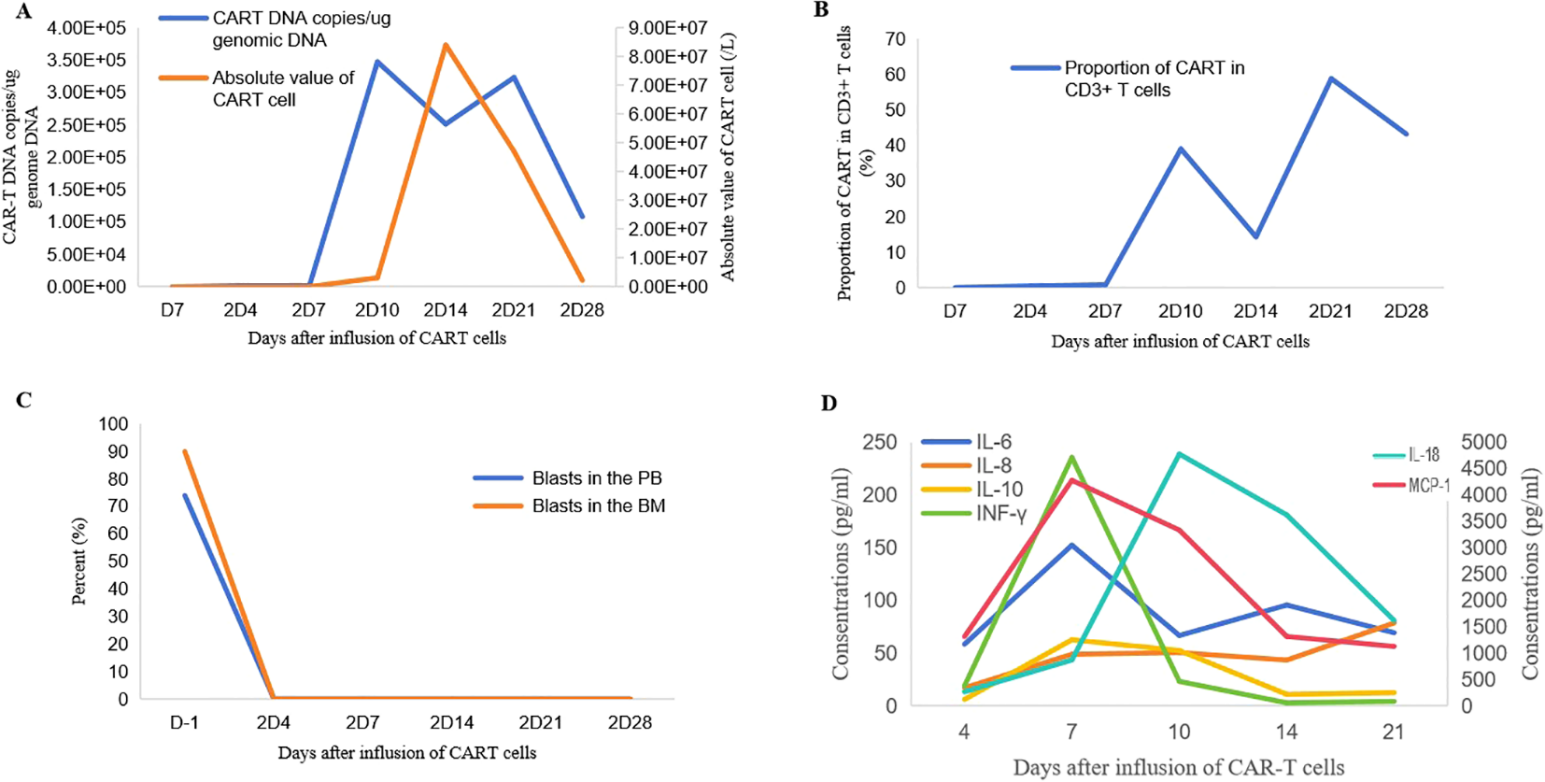
Changes in case 2 indicators in case 2 after CD7 CAR T-cell therapy. Trends in CAR T DNA copies and absolute value of CAR T-cell counts in PB **(A)**. Proportion of CAR T cells among CD3^+^ T cells **(B)**. Blasts in BM and PB **(C)**. Levels of inflammatory cytokines **(D)**. 2D, days after the second CAR T-cell infusion.

After intrathecal injection and FC regimen preconditioning, the patient received an infusion of autologous humanized NS7CAR T cells (Hebei Senlangio Technology Co., Ltd., Shijiazhuang, China). The dose of the effective CD7 CAR T cells in the infusion was 5 × 10^5^ cells/kg. The WBC count in peripheral blood (PB) briefly declined and then rapidly increased to 425 × 10^9^/L soon on day 3 after CAR T-cell infusion, indicating ongoing disease progression. Cyclophosphamide, dexamethasone, and idarubicin were administered for tumor burden reduction. Subsequently, the patient received second CD7 CAR T-cell infusion, with an effective dose of 2.5 × 10^5^ cells/kg. As observed in case 1, approximately two weeks later, the absolute number of CAR T cells reached a peak (**Figures 4A, B**), and the patient achieved complete remission (CR) with minimal residual disease negativity (MRD−) in the bone marrow (BM), as verified by flow cytometry (FCM) (**Figure 4C**).

Grade 1 cytokine release syndrome (CRS), manifested by fever and muscle aches, was observed after CD7 CAR T-cell infusion and was successfully managed. CAR T-cell–related encephalopathy syndrome (CRES) events were not detected during therapy. Several inflammatory biomarkers, including interleukin-18 (IL-18) and monocyte chemoattractant protein-1 (MCP-1), peaked approximately 1–2 weeks after the CD7 CAR T-cell infusion (**Figure 4D**), likely associated with CRS. Neutrophil counts exceeded 0.5 × 10^9^/L on day 41. Red blood cell and platelet transfusion dependence persisted prior to allo-HSCT (**Supplementary Figure S2**).

On day 78 after the second CAR T-cell therapy, the patient received myeloablative conditioning consisting of total body irradiation (TBI), cytarabine, cladribine, and idarubicin (**Figure 3**). Anti-thymocyte globulin (ATG), cyclosporine A and methotrexate were administered for GVHD prophylaxis. The patient then underwent infusion of allogeneic hematopoietic stem cells from his father on July 6, 2021 during the second CR. Neutrophil and platelet engraftment occurred on days +12 and +13, respectively. Short tandem repeat (STR) analysis using fluorescence-labeled polymerase chain reaction (PCR) demonstrated complete donor chimerism on day +30.

On day +37 after allo-HSCT, the patient was readmitted because of headache and fever. Epstein–Barr virus (EBV) DNA was detected in the PB and cerebrospinal fluid (CSF) at levels of 5.19 × 10^5^ copies/mL and 2.76 × 10³ copies/mL, respectively. Concurrently, multiple enlarged lymph nodes were observed in the bilateral cervical, supraclavicular, axillary, and inguinal regions. Brain magnetic resonance imaging (MRI) revealed small patchy hyperintense lesions near the posterior horn of the right lateral ventricle on T2-weighted images (**Supplementary Figure S3**). Based on these findings, the patient was diagnosed with EBV disease, EBV encephalitis, and probable EBV-associated post-transplant lymphoproliferative disease (EBV-PTLD). Glucocorticoids were reduced rapidly tapered, and rituximab was administered via intrathecal injection (40 mg, three doses) and intravenous infusion (375 mg/m² weekly, three doses). Clinical symptoms resolved completely, and EBV-DNA became undetectable after the first rituximab administration. Two weeks later, follow-up brain MRI showed a marked reduction in lesion size of the lesions.

Subsequently, skin rash and diarrhea developed on day +97 after allo-HSCT. The dosage of cyclosporine A was appropriately adjusted based on serum concentration monitoring. On day +104 after allo-HSCT, the patient developed afebrile convulsions accompanied by dizziness and headache. The serum of cyclosporine A at that time was 406 ng/mL. Brain MRI demonstrated resolution of the previous periventricular lesions near the right ventricle disappeared, but revealed new patchy lesions in the bilateral frontal and parietal cortices, consistent with reversible posterior encephalopathy syndrome (**Supplementary Figure S3**). Cyclosporine A was discontinued, and glucocorticoids and mycophenolate mofetil (MMF) were administered for treating GVHD management. The patient’s clinical symptoms resolved completely following treatment.

On day +184, CSF analysis revealed leukemic cells, indicating central nervous system leukemia (CNSL). Residual leukemic cells in the CSF became undetectable after two courses of intrathecal chemotherapy. Oral venetoclax (100 mg daily) was subsequently initiated to prevent disease recurrence. However, on day +388, abnormal T cells accounted for 54.0% in the CSF and 23.0% in the BM. Leukemic cells initially decreased but later increased, and the patient has developed progressive central nervous system symptoms, primarily manifesting with paraplegia, despite receiving further anti-leukemia treatments, including azacitidine (100 mg subcutaneously on days 1–7) combined with venetoclax (400 mg orally on days 1–14), CD7 CAR T-cell infusion, hematopoietic stem cell infusion from his father, and multiple courses of intrathecal chemotherapy. Ultimately, treatment was discontinued for financial reasons, and the patient eventually died of leukemia on day +561.

## Discussion

This case report describes two patients who collectively experienced nearly all the major challenging complications across the CAR T–to–allo-HSCT treatment continuum. Case 1 exemplifies a critical trajectory characterized by severe inflammation, cumulative toxicity, and a cascade of serious complications; in contrast, case 2 demonstrated a relatively favorable course marked by effective inflammation control, complete recovery following CAR T-cell infusion, and timely subsequent transplantation. These findings highlight that the systemic inflammatory status and immune environment following CAR T therapy influence the outcome of the subsequent allo-HSCT greatly, with the timing of the bridging intervention acting as a pivotal and clinically modifiable factor.

According to the diagnostic criteria proposed by Cho et al. (13), case 1 was considered to have transplant-associated thrombotic microangiopathy (TA-TMA). The pathogenesis of TA-TMA is primarily driven by endothelial cell injury, which triggers activation of the complement system, leading to platelet aggregation and microvascular thrombosis (14, 15). Evidences from previous studies indicate that endothelial cell damage can be induced by CAR T-cell therapy, conditioning regimens, and GVHD (16–18). Therefore, the adverse outcome observed in case 1 was not an accidental phenomenon but rather reflects a prototypical “multiple-hit” pathophysiological model. Grade 3 CRS, together with bone marrow suppression and infection associated with higher-dose of CAR T-cell therapy (19), constituted the initial major hits. Drug-related organ toxicity during the pretransplant conditioning phase represented the second significant insult, exemplified by agents such as cyclosporine A. GVHD following donor cell infusion served as the third critical hit. The cumulative effects of these insults led to progressive endothelial cell injury, ultimately triggering TA-TMA. This process may initiate a vicious cycle between GVHD and TA-TMA, potentially resulting in multiple organ failure. Recent clinical studies have also reported that CAR T-cell therapy prior to HSCT is a risk factor for moderate to severe chronic GVHD and TA-TMA (20, 21). We hypothesize that the unresolved inflammatory status associated with the CRS after CAR T therapy may contribute to or exacerbate TA-TMA following allo-HSCT, although there are currently limited data on whether the severity of CAR T-cell–related toxicities (CRS/CRES) correlates with post-HSCT complications. Although case 1 received first-line and second-line treatments for TMA (22), the patient ultimately died from this complication.

In contrast, case 2 successfully avoided this life-threatening cascade, which may be attributable to only mild inflammation above the baseline after CD7 CAR T therapy and a prolonged recovery interval of nearly three months before allo-HSCT. Although radiotherapy, CAR T therapy, venetoclax, and HSCT were administered in accordance with studies indicating the potential therapeutic benefits for central nervous system leukemia (CNSL) (23–26), the patient ultimately succumbed to disease recurrence.

The optimal interval between CAR T-cell therapy and subsequent HSCT represents a critical, clinically modifiable factor. Although the time interval varied between studies, the median interval time from CAR T therapy to consolidative allo-HSCT is generally approximately 50–90 days (27). Cao et al. (28) reported no significant differences in outcomes between intervals of >30 but <60 days and >60 days. However, evidences from previous clinical trials indicate that, without allo-HSCT, high-risk patients tend to experience early relapse after CAR T therapy (8, 9). Therefore, consolidative allo-HSCT within 90 days may be considered to reduce the risk of early relapse, particularly in high-risk patients. Nevertheless, if a persistent inflammatory cytokine response or endothelial injury is observed following CAR T therapy, we recommend delaying HSCT until full recovery from the inflammation. Thus, the interval between the bridging CAR T-cell therapy and subsequent allo-HSCT should be optimized based on the patient’s inflammatory status and levels of inflammatory mediators.

Selection of conditioning regimens represents another important clinical challenge. For patients at high risk of relapse after allo-HSCT, myeloablative conditioning regimens may improve disease-free survival (29). Furthermore, a recent retrospective study showed that reduced-intensity conditioning (RIC) may represent a safer option, with 1-year event-free survival (EFS) rate of 73% (27). Some clinicians have even suggested that CAR T-cell therapy could be incorporated as a part of the conditioning regimen prior to HSCT (30). In addition, Hu et al. tested a novel “all-in-one” strategy consisting of sequential CD7 CAR T-cell therapy followed by haploidentical HSCT in 10 patients with relapsed or refractory CD7-positive leukemia or lymphoma (31). Determination of the optimal interval between CAR T-cell therapy and allo-HSCT, as well as selection of the conditioning regimen, should be based on an individualized assessment of the patient’s physical condition, disease status, comorbidities, levels of inflammatory markers, and organ function.

In summary, CD7 CAR T-cell therapy offers a promising treatment option for R/R T-ALL. However, its integration with allo-HSCT represents a potentially effective yet high-risk clinical strategy and therefore requires careful and individualized management. Implementation of this approach should also involve balancing potential health benefits against associated costs. The two cases presented here underscore the limitations of rigid paradigms regarding bridging timing and conditioning intensity. An effective therapeutic strategy must remain adaptive, taking into account the dynamic immune alterations induced by CAR T-cell therapy. Future prospective studies are urgently needed to develop robust predictive models that can guide clinical decision-making in this complex therapeutic landscape.

## Limitations of this report

As the represented data derived from only two case reports, our conclusions are primarily hypothesis-generating and do not provide definitive evidence regarding the optimal interval or conditioning regimen. Additional data from larger patient cohorts are therefore warranted. Furthermore, whether the severity of CAR T-cell–induced toxicities (CRS/CRES) correlates with complications following allo-HSCT remains unclear. To obtain more robust evidence supporting this combined therapeutic strategy, rigorous clinical management and more detailed biomarker analyses will be required.

## Data Availability

The original contributions presented in the study are included in the article/**Supplementary Material**. Further inquiries can be directed to the corresponding author.
