# Supplementary material for: Case report: Different outcomes of two cases of relapsed/refractory T cell acute lymphoblastic leukemia treated with anti-CD7 chimeric antigen receptor T cells bridging to allogeneic hematopoietic stem cell transplantation: from curative promise to fatal risk
Source: Front Oncol. 2026 Jan 29;16:1766948. doi: 10.3389/fonc.2026.1766948 (PMC12894014; doi:10.3389/fonc.2026.1766948)
Supplement: Supplementary file 1 [file Presentation1.pptx]

## Slide 1
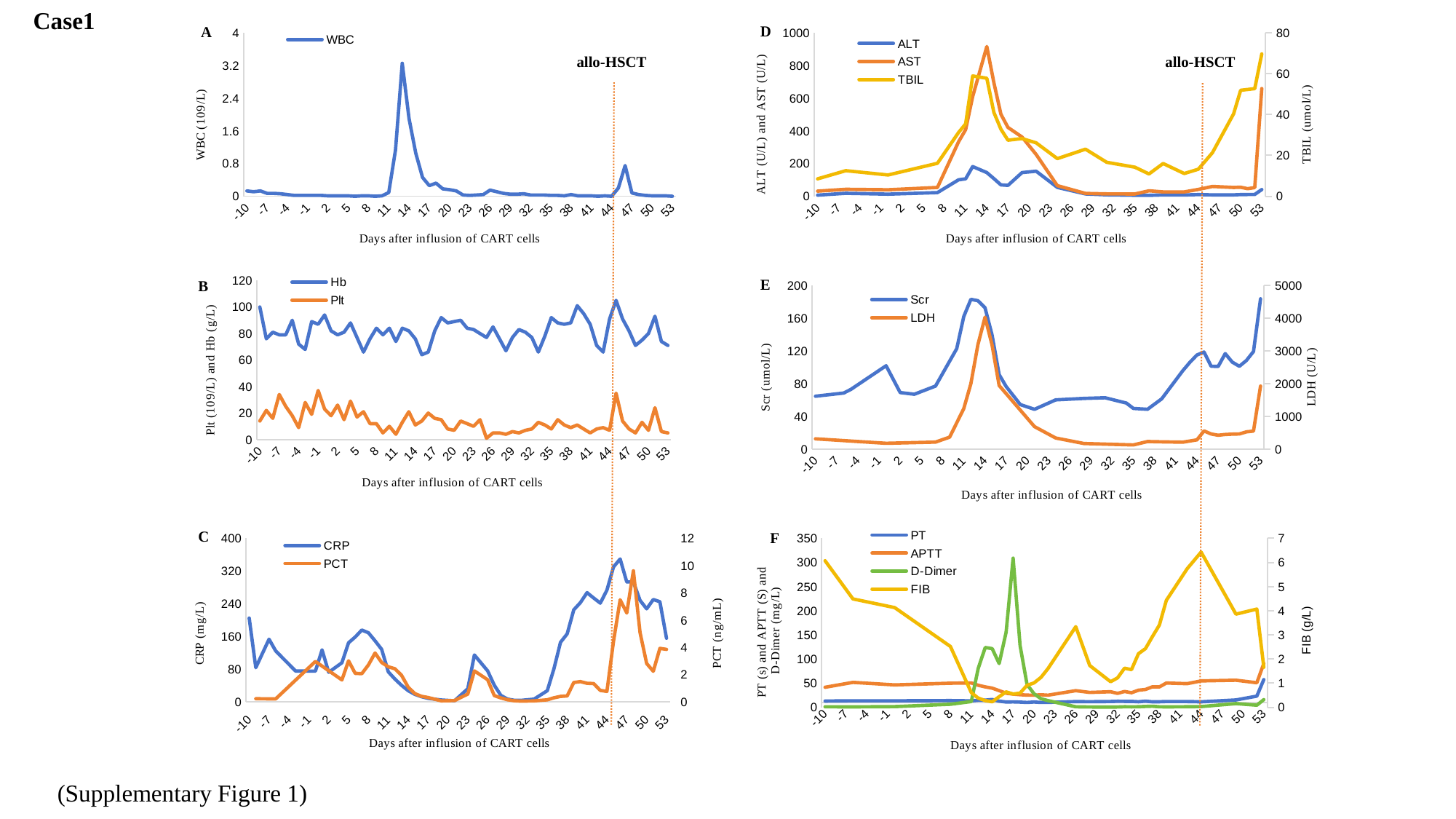

Case1
### Chart
| Category | WBC |
|---|---|
| -10 | 0.13 |
| -9 | 0.11 |
| -8 | 0.13 |
| -7 | 0.07 |
| -6 | 0.07 |
| -5 | 0.06 |
| -4 | 0.04 |
| -3 | 0.02 |
| -2 | 0.02 |
| -1 | 0.02 |
| 0 | 0.02 |
| 1 | 0.02 |
| 2 | 0.01 |
| 3 | 0.01 |
| 4 | 0.01 |
| 5 | 0.01 |
| 6 | 0.0 |
| 7 | 0.01 |
| 8 | 0.01 |
| 9 | 0.0 |
| 10 | 0.01 |
| 11 | 0.09 |
| 12 | 1.13 |
| 13 | 3.25 |
| 14 | 1.9 |
| 15 | 1.05 |
| 16 | 0.46 |
| 17 | 0.26 |
| 18 | 0.32 |
| 19 | 0.18 |
| 20 | 0.16 |
| 21 | 0.13 |
| 22 | 0.03 |
| 23 | 0.02 |
| 24 | 0.03 |
| 25 | 0.04 |
| 26 | 0.15 |
| 27 | 0.11 |
| 28 | 0.07 |
| 29 | 0.05 |
| 30 | 0.05 |
| 31 | 0.06 |
| 32 | 0.03 |
| 33 | 0.03 |
| 34 | 0.03 |
| 35 | 0.02 |
| 36 | 0.02 |
| 37 | 0.01 |
| 38 | 0.04 |
| 39 | 0.01 |
| 40 | 0.01 |
| 41 | 0.01 |
| 42 | 0.0 |
| 43 | 0.01 |
| 44 | 0.0 |
| 45 | 0.2 |
| 46 | 0.75 |
| 47 | 0.08 |
| 48 | 0.04 |
| 49 | 0.02 |
| 50 | 0.01 |
| 51 | 0.01 |
| 52 | 0.01 |
| 53 | 0.0 |
### Chart
| Category | ALT | AST | TBIL |
|---|---|---|---|D
A
allo-HSCT
allo-HSCT
### Chart
| Category | Hb | Plt |
|---|---|---|
| -10 | 100.0 | 14.0 |
| -9 | 76.0 | 22.0 |
| -8 | 81.0 | 16.0 |
| -7 | 79.0 | 34.0 |
| -6 | 79.0 | 25.0 |
| -5 | 90.0 | 18.0 |
| -4 | 72.0 | 9.0 |
| -3 | 68.0 | 28.0 |
| -2 | 89.0 | 19.0 |
| -1 | 87.0 | 37.0 |
| 0 | 94.0 | 23.0 |
| 1 | 82.0 | 18.0 |
| 2 | 79.0 | 26.0 |
| 3 | 81.0 | 15.0 |
| 4 | 88.0 | 29.0 |
| 5 | 77.0 | 17.0 |
| 6 | 66.0 | 21.0 |
| 7 | 76.0 | 12.0 |
| 8 | 84.0 | 12.0 |
| 9 | 79.0 | 5.0 |
| 10 | 84.0 | 10.0 |
| 11 | 74.0 | 4.0 |
| 12 | 84.0 | 13.0 |
| 13 | 82.0 | 21.0 |
| 14 | 76.0 | 11.0 |
| 15 | 64.0 | 14.0 |
| 16 | 66.0 | 20.0 |
| 17 | 82.0 | 16.0 |
| 18 | 92.0 | 15.0 |
| 19 | 88.0 | 8.0 |
| 20 | 89.0 | 7.0 |
| 21 | 90.0 | 14.0 |
| 22 | 84.0 | 12.0 |
| 23 | 83.0 | 10.0 |
| 24 | 80.0 | 15.0 |
| 25 | 77.0 | 1.0 |
| 26 | 85.0 | 5.0 |
| 27 | 76.0 | 5.0 |
| 28 | 67.0 | 4.0 |
| 29 | 77.0 | 6.0 |
| 30 | 83.0 | 5.0 |
| 31 | 81.0 | 7.0 |
| 32 | 77.0 | 8.0 |
| 33 | 66.0 | 13.0 |
| 34 | 78.0 | 11.0 |
| 35 | 92.0 | 8.0 |
| 36 | 88.0 | 15.0 |
| 37 | 87.0 | 11.0 |
| 38 | 88.0 | 9.0 |
| 39 | 101.0 | 11.0 |
| 40 | 95.0 | 8.0 |
| 41 | 87.0 | 5.0 |
| 42 | 71.0 | 8.0 |
| 43 | 66.0 | 9.0 |
| 44 | 91.0 | 7.0 |
| 45 | 105.0 | 35.0 |
| 46 | 91.0 | 14.0 |
| 47 | 82.0 | 8.0 |
| 48 | 71.0 | 5.0 |
| 49 | 75.0 | 13.0 |
| 50 | 80.0 | 7.0 |
| 51 | 93.0 | 24.0 |
| 52 | 74.0 | 6.0 |
| 53 | 71.0 | 5.0 |
### Chart
| Category | Scr | LDH |
|---|---|---|
| -10 | 64.6 | 313.2 |
| -9 | None | None |
| -8 | None | None |
| -7 | None | None |
| -6 | 68.5 | None |
| -5 | 72.9 | None |
| -4 | None | None |
| -3 | None | None |
| -2 | None | None |
| -1 | None | None |
| 0 | 101.9 | 176.2 |
| 1 | None | None |
| 2 | 69.05 | None |
| 3 | None | None |
| 4 | 67.0 | None |
| 5 | None | None |
| 6 | None | None |
| 7 | 77.06 | 213.71 |
| 8 | None | None |
| 9 | None | 364.27 |
| 10 | 122.8 | None |
| 11 | 162.1 | 1237.78 |
| 12 | 183.0 | 1998.94 |
| 13 | 181.38 | 3192.6 |
| 14 | 172.74 | 4031.72 |
| 15 | 139.43 | 3180.2 |
| 16 | 91.09 | 1943.63 |
| 17 | 76.4 | None |
| 18 | None | None |
| 19 | 54.37 | None |
| 20 | None | None |
| 21 | 48.59 | 686.21 |
| 22 | None | None |
| 23 | None | None |
| 24 | 60.1 | 338.2 |
| 25 | None | None |
| 26 | None | None |
| 27 | None | None |
| 28 | 61.92 | 170.73 |
| 29 | None | None |
| 30 | None | None |
| 31 | 62.7 | None |
| 32 | None | None |
| 33 | None | None |
| 34 | 56.25 | None |
| 35 | 49.65 | 127.24 |
| 36 | None | None |
| 37 | 48.64 | 231.0 |
| 38 | None | None |
| 39 | 61.32 | 220.93 |
| 40 | None | None |
| 41 | None | None |
| 42 | 95.87 | 210.92 |
| 43 | 106.1 | None |
| 44 | 115.0 | 281.7 |
| 45 | 118.6 | 554.3 |
| 46 | 101.3 | 461.82 |
| 47 | 101.0 | 422.35 |
| 48 | 116.71 | 445.83 |
| 49 | 106.28 | 455.84 |
| 50 | 101.2 | 462.3 |
| 51 | 108.3 | 525.5 |
| 52 | 119.3 | 549.7 |
| 53 | 183.68 | 1926.59 |E
B
### Chart
| Category | CRP | PCT |
|---|---|---|C
### Chart
| Category | PT | APTT | D-Dimer | FIB |
|---|---|---|---|---|F
(Supplementary Figure 1)

## Slide 2
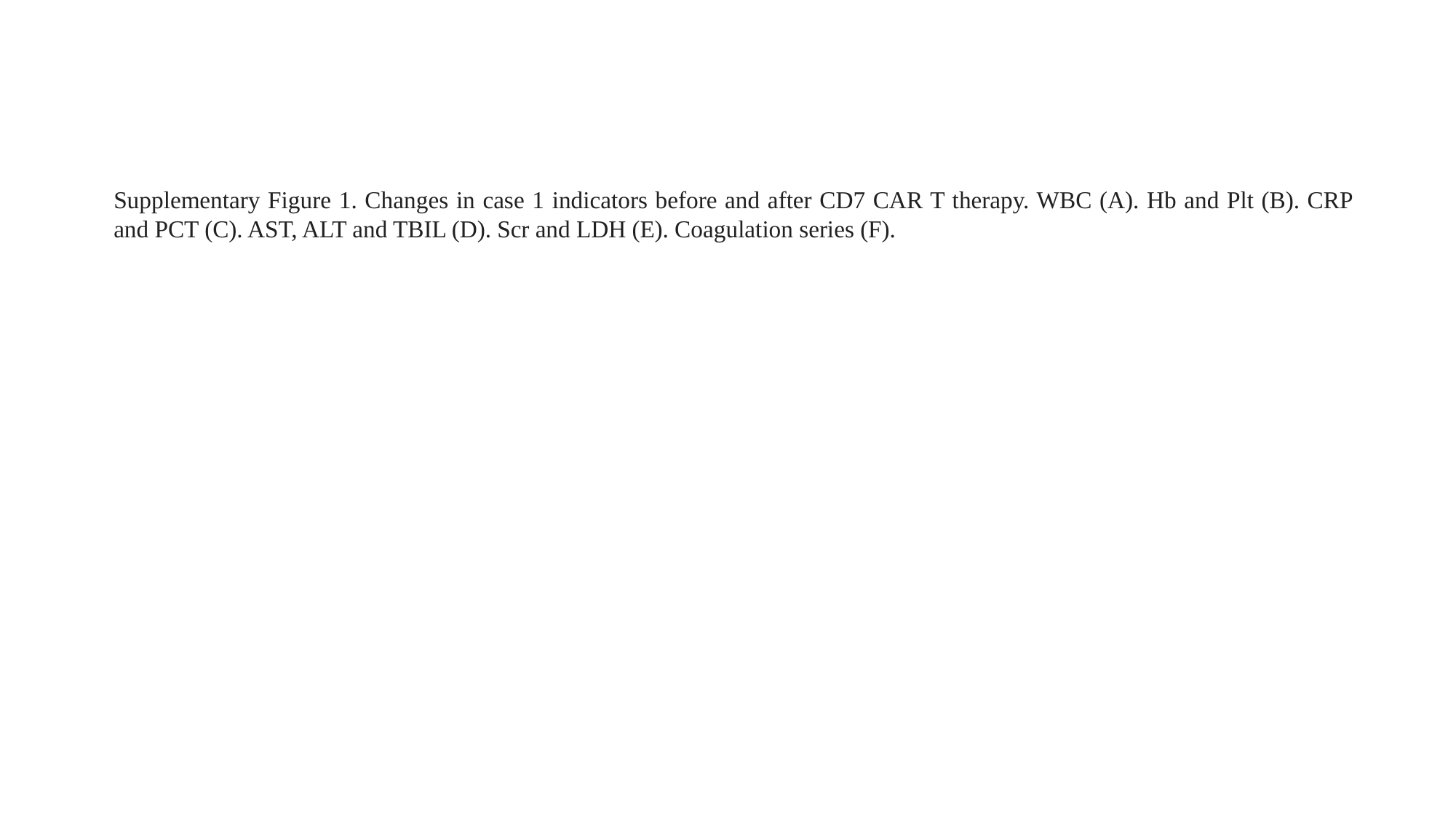

Supplementary Figure 1. Changes in case 1 indicators before and after CD7 CAR T therapy. WBC (A). Hb and Plt (B). CRP and PCT (C). AST, ALT and TBIL (D). Scr and LDH (E). Coagulation series (F).

## Slide 3
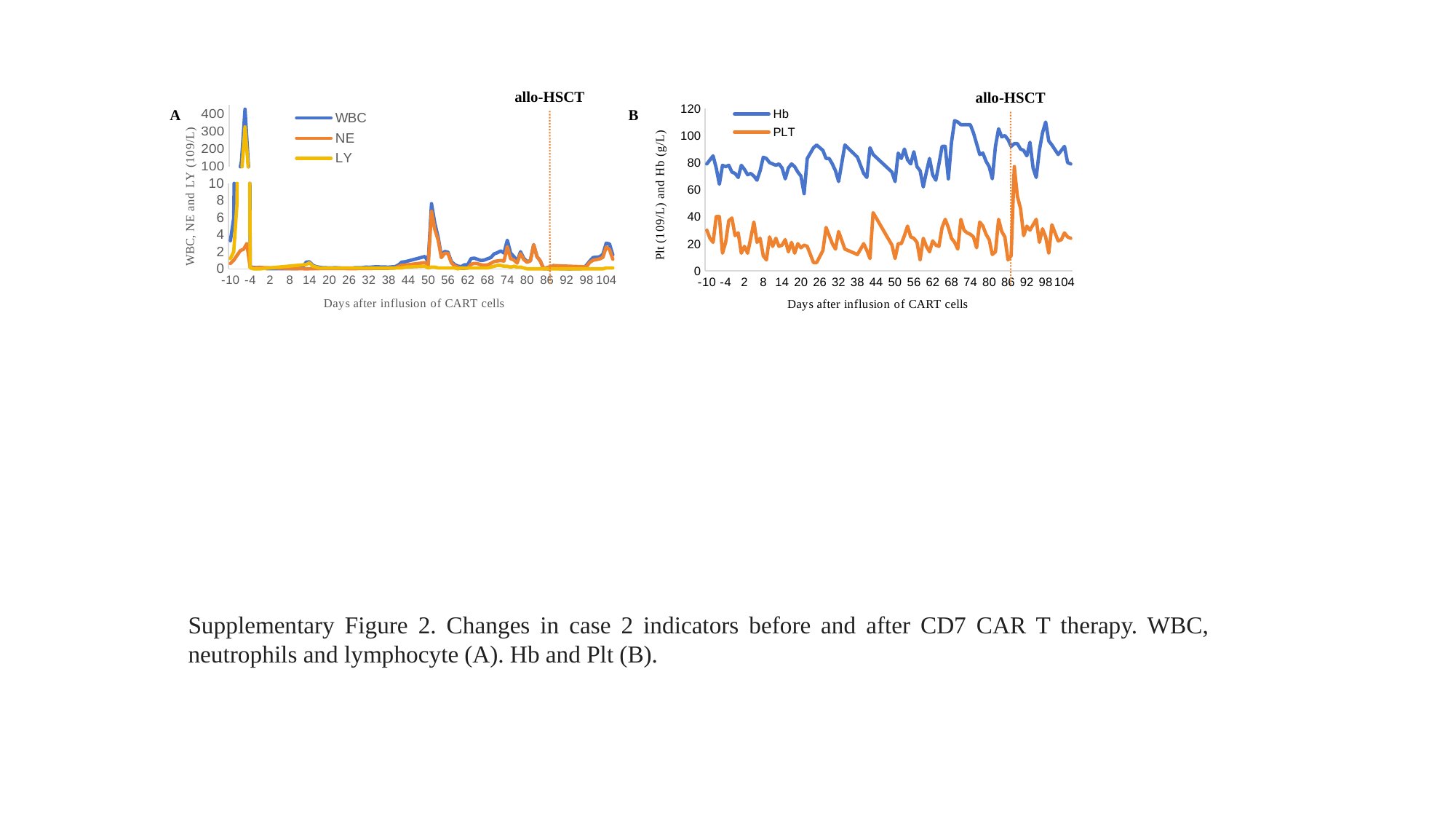

### Chart
| Category | WBC | NE | LY |
|---|---|---|---|
| -10 | 3.28 | 0.62 | 1.2 |
| -9 | 5.92 | 1.0 | 2.0 |
| -8 | 35.58 | 1.56 | 7.6 |
| -7 | 132.19 | 2.12 | 53.7 |
| -6 | 425.79 | 2.29 | 325.7 |
| -5 | 100.2 | 2.95 | 77.5 |
| -4 | 0.24 | 0.15 | 0.1 |
| -3 | 0.16 | 0.12 | 0.0 |
| -2 | 0.13 | 0.12 | 0.0 |
| -1 | 0.18 | 0.15 | 0.0 |
| 0 | 0.08 | None | None |
| 1 | 0.02 | None | None |
| 2 | 0.01 | None | None |
| 3 | 0.01 | None | None |
| 4 | 0.01 | None | None |
| 5 | 0.03 | None | None |
| 6 | 0.01 | None | None |
| 7 | 0.01 | None | None |
| 8 | 0.01 | None | None |
| 9 | 0.01 | None | None |
| 10 | 0.01 | None | None |
| 11 | 0.07 | None | None |
| 12 | 0.24 | None | None |
| 13 | 0.78 | 0.0 | 0.5 |
| 14 | 0.81 | 0.01 | 0.7 |
| 15 | 0.41 | 0.01 | 0.3 |
| 16 | 0.25 | 0.01 | 0.2 |
| 17 | 0.19 | 0.03 | 0.1 |
| 18 | 0.13 | 0.04 | 0.1 |
| 19 | 0.13 | 0.04 | 0.1 |
| 20 | 0.1 | None | None |
| 21 | 0.12 | 0.03 | 0.1 |
| 22 | 0.13 | 0.04 | 0.1 |
| 23 | None | None | None |
| 24 | 0.09 | None | None |
| 25 | 0.09 | None | None |
| 26 | None | None | None |
| 27 | 0.09 | None | None |
| 28 | 0.14 | 0.02 | 0.1 |
| 29 | 0.14 | 0.04 | 0.1 |
| 30 | 0.14 | 0.03 | 0.1 |
| 31 | 0.2 | 0.05 | 0.1 |
| 32 | 0.18 | 0.04 | 0.1 |
| 33 | None | None | None |
| 34 | 0.24 | 0.06 | 0.1 |
| 35 | None | None | None |
| 36 | None | None | None |
| 37 | None | None | None |
| 38 | 0.2 | 0.07 | 0.1 |
| 39 | None | None | None |
| 40 | 0.26 | 0.1 | 0.1 |
| 41 | 0.46 | 0.28 | 0.1 |
| 42 | 0.79 | 0.42 | 0.1 |
| 43 | 0.82 | 0.43 | 0.2 |
| 44 | None | None | None |
| 45 | None | None | None |
| 46 | None | None | None |
| 47 | None | None | None |
| 48 | None | None | None |
| 49 | 1.44 | 0.72 | 0.3 |
| 50 | 0.81 | 0.43 | 0.1 |
| 51 | 7.62 | 6.72 | 0.2 |
| 52 | 5.32 | 4.61 | 0.2 |
| 53 | 3.72 | 3.38 | 0.1 |
| 54 | 1.53 | 1.31 | 0.1 |
| 55 | 2.03 | 1.82 | 0.1 |
| 56 | 1.95 | 1.79 | 0.1 |
| 57 | 0.86 | 0.71 | 0.1 |
| 58 | 0.5 | 0.35 | 0.1 |
| 59 | 0.32 | 0.18 | 0.0 |
| 60 | 0.25 | 0.05 | 0.1 |
| 61 | 0.47 | 0.07 | 0.1 |
| 62 | 0.48 | 0.08 | 0.1 |
| 63 | 1.2 | 0.53 | 0.1 |
| 64 | 1.25 | 0.63 | 0.1 |
| 65 | 1.11 | 0.6 | 0.1 |
| 66 | 0.97 | 0.47 | 0.1 |
| 67 | 1.02 | 0.43 | 0.1 |
| 68 | 1.18 | 0.45 | 0.1 |
| 69 | 1.31 | 0.63 | 0.2 |
| 70 | 1.74 | 0.85 | 0.3 |
| 71 | 1.89 | 0.92 | 0.4 |
| 72 | 2.09 | 0.97 | 0.4 |
| 73 | 1.85 | 0.9 | 0.3 |
| 74 | 3.34 | 2.59 | 0.3 |
| 75 | 1.84 | 1.18 | 0.2 |
| 76 | 1.43 | 1.03 | 0.3 |
| 77 | 0.91 | 0.67 | 0.2 |
| 78 | 2.0 | 1.77 | 0.2 |
| 79 | 1.23 | 1.08 | 0.1 |
| 80 | 0.83 | 0.76 | 0.0 |
| 81 | 0.96 | 0.93 | 0.0 |
| 82 | 2.83 | 2.81 | 0.0 |
| 83 | 1.44 | 1.43 | 0.0 |
| 84 | 0.92 | 0.92 | 0.0 |
| 85 | 0.02 | 0.02 | 0.0 |
| 86 | 0.01 | None | None |
| 87 | 0.0 | None | None |
| 88 | 0.38 | 0.37 | 0.0 |
| 89 | 0.06 | None | None |
| 90 | 0.03 | None | None |
| 91 | 0.01 | None | None |
| 92 | 0.01 | None | None |
| 93 | 0.0 | None | None |
| 94 | 0.02 | None | None |
| 95 | 0.01 | None | None |
| 96 | 0.02 | None | None |
| 97 | 0.06 | None | None |
| 98 | 0.41 | 0.23 | 0.0 |
| 99 | 0.9 | 0.75 | 0.0 |
| 100 | 1.32 | 1.0 | 0.0 |
| 101 | None | None | None |
| 102 | 1.4 | 1.16 | 0.0 |
| 103 | 1.7 | 1.34 | 0.0 |
| 104 | 3.0 | 2.54 | 0.1 |
| 105 | 2.91 | 2.26 | 0.1 |
| 106 | 1.63 | 1.12 | 0.1 |allo-HSCT
allo-HSCT
### Chart
| Category | WBC | NE | LY |
|---|---|---|---|
| -10 | 3.28 | 0.62 | 1.2 |
| -9 | 5.92 | 1.0 | 2.0 |
| -8 | 35.58 | 1.56 | 7.6 |
| -7 | 132.19 | 2.12 | 53.7 |
| -6 | 425.79 | 2.29 | 325.7 |
| -5 | 100.2 | 2.95 | 77.5 |
| -4 | 0.24 | 0.15 | 0.1 |
| -3 | 0.16 | 0.12 | 0.0 |
| -2 | 0.13 | 0.12 | 0.0 |
| -1 | 0.18 | 0.15 | 0.0 |
| 0 | 0.08 | None | None |
| 1 | 0.02 | None | None |
| 2 | 0.01 | None | None |
| 3 | 0.01 | None | None |
| 4 | 0.01 | None | None |
| 5 | 0.03 | None | None |
| 6 | 0.01 | None | None |
| 7 | 0.01 | None | None |
| 8 | 0.01 | None | None |
| 9 | 0.01 | None | None |
| 10 | 0.01 | None | None |
| 11 | 0.07 | None | None |
| 12 | 0.24 | None | None |
| 13 | 0.78 | 0.0 | 0.5 |
| 14 | 0.81 | 0.01 | 0.7 |
| 15 | 0.41 | 0.01 | 0.3 |
| 16 | 0.25 | 0.01 | 0.2 |
| 17 | 0.19 | 0.03 | 0.1 |
| 18 | 0.13 | 0.04 | 0.1 |
| 19 | 0.13 | 0.04 | 0.1 |
| 20 | 0.1 | None | None |
| 21 | 0.12 | 0.03 | 0.1 |
| 22 | 0.13 | 0.04 | 0.1 |
| 23 | None | None | None |
| 24 | 0.09 | None | None |
| 25 | 0.09 | None | None |
| 26 | None | None | None |
| 27 | 0.09 | None | None |
| 28 | 0.14 | 0.02 | 0.1 |
| 29 | 0.14 | 0.04 | 0.1 |
| 30 | 0.14 | 0.03 | 0.1 |
| 31 | 0.2 | 0.05 | 0.1 |
| 32 | 0.18 | 0.04 | 0.1 |
| 33 | None | None | None |
| 34 | 0.24 | 0.06 | 0.1 |
| 35 | None | None | None |
| 36 | None | None | None |
| 37 | None | None | None |
| 38 | 0.2 | 0.07 | 0.1 |
| 39 | None | None | None |
| 40 | 0.26 | 0.1 | 0.1 |
| 41 | 0.46 | 0.28 | 0.1 |
| 42 | 0.79 | 0.42 | 0.1 |
| 43 | 0.82 | 0.43 | 0.2 |
| 44 | None | None | None |
| 45 | None | None | None |
| 46 | None | None | None |
| 47 | None | None | None |
| 48 | None | None | None |
| 49 | 1.44 | 0.72 | 0.3 |
| 50 | 0.81 | 0.43 | 0.1 |
| 51 | 7.62 | 6.72 | 0.2 |
| 52 | 5.32 | 4.61 | 0.2 |
| 53 | 3.72 | 3.38 | 0.1 |
| 54 | 1.53 | 1.31 | 0.1 |
| 55 | 2.03 | 1.82 | 0.1 |
| 56 | 1.95 | 1.79 | 0.1 |
| 57 | 0.86 | 0.71 | 0.1 |
| 58 | 0.5 | 0.35 | 0.1 |
| 59 | 0.32 | 0.18 | 0.0 |
| 60 | 0.25 | 0.05 | 0.1 |
| 61 | 0.47 | 0.07 | 0.1 |
| 62 | 0.48 | 0.08 | 0.1 |
| 63 | 1.2 | 0.53 | 0.1 |
| 64 | 1.25 | 0.63 | 0.1 |
| 65 | 1.11 | 0.6 | 0.1 |
| 66 | 0.97 | 0.47 | 0.1 |
| 67 | 1.02 | 0.43 | 0.1 |
| 68 | 1.18 | 0.45 | 0.1 |
| 69 | 1.31 | 0.63 | 0.2 |
| 70 | 1.74 | 0.85 | 0.3 |
| 71 | 1.89 | 0.92 | 0.4 |
| 72 | 2.09 | 0.97 | 0.4 |
| 73 | 1.85 | 0.9 | 0.3 |
| 74 | 3.34 | 2.59 | 0.3 |
| 75 | 1.84 | 1.18 | 0.2 |
| 76 | 1.43 | 1.03 | 0.3 |
| 77 | 0.91 | 0.67 | 0.2 |
| 78 | 2.0 | 1.77 | 0.2 |
| 79 | 1.23 | 1.08 | 0.1 |
| 80 | 0.83 | 0.76 | 0.0 |
| 81 | 0.96 | 0.93 | 0.0 |
| 82 | 2.83 | 2.81 | 0.0 |
| 83 | 1.44 | 1.43 | 0.0 |
| 84 | 0.92 | 0.92 | 0.0 |
| 85 | 0.02 | 0.02 | 0.0 |
| 86 | 0.01 | None | None |
| 87 | 0.0 | None | None |
| 88 | 0.38 | 0.37 | 0.0 |
| 89 | 0.06 | None | None |
| 90 | 0.03 | None | None |
| 91 | 0.01 | None | None |
| 92 | 0.01 | None | None |
| 93 | 0.0 | None | None |
| 94 | 0.02 | None | None |
| 95 | 0.01 | None | None |
| 96 | 0.02 | None | None |
| 97 | 0.06 | None | None |
| 98 | 0.41 | 0.23 | 0.0 |
| 99 | 0.9 | 0.75 | 0.0 |
| 100 | 1.32 | 1.0 | 0.0 |
| 101 | None | None | None |
| 102 | 1.4 | 1.16 | 0.0 |
| 103 | 1.7 | 1.34 | 0.0 |
| 104 | 3.0 | 2.54 | 0.1 |
| 105 | 2.91 | 2.26 | 0.1 |
| 106 | 1.63 | 1.12 | 0.1 |
### Chart
| Category | Hb | PLT |
|---|---|---|A
B
Supplementary Figure 2. Changes in case 2 indicators before and after CD7 CAR T therapy. WBC, neutrophils and lymphocyte (A). Hb and Plt (B).

## Slide 4
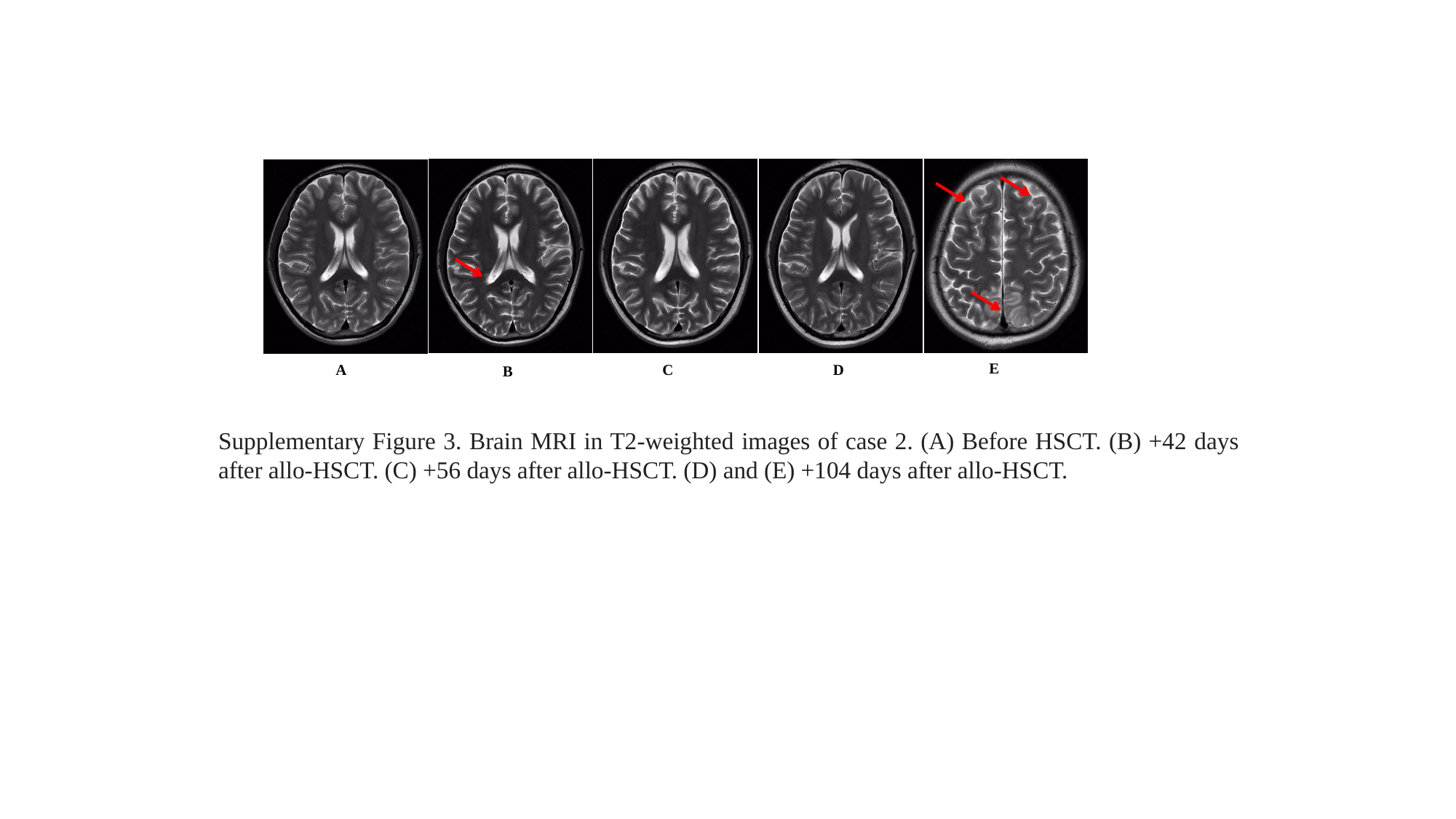

E
D
A
C
B
Supplementary Figure 3. Brain MRI in T2-weighted images of case 2. (A) Before HSCT. (B) +42 days after allo-HSCT. (C) +56 days after allo-HSCT. (D) and (E) +104 days after allo-HSCT.
